# Supplementary figures and images for: Structure and evolution of alanine/serine decarboxylases and the engineering of theanine production
Source: eLife. 2024 Sep 17;12:RP91046. doi: 10.7554/eLife.91046 (PMC11407765; doi:10.7554/eLife.91046)

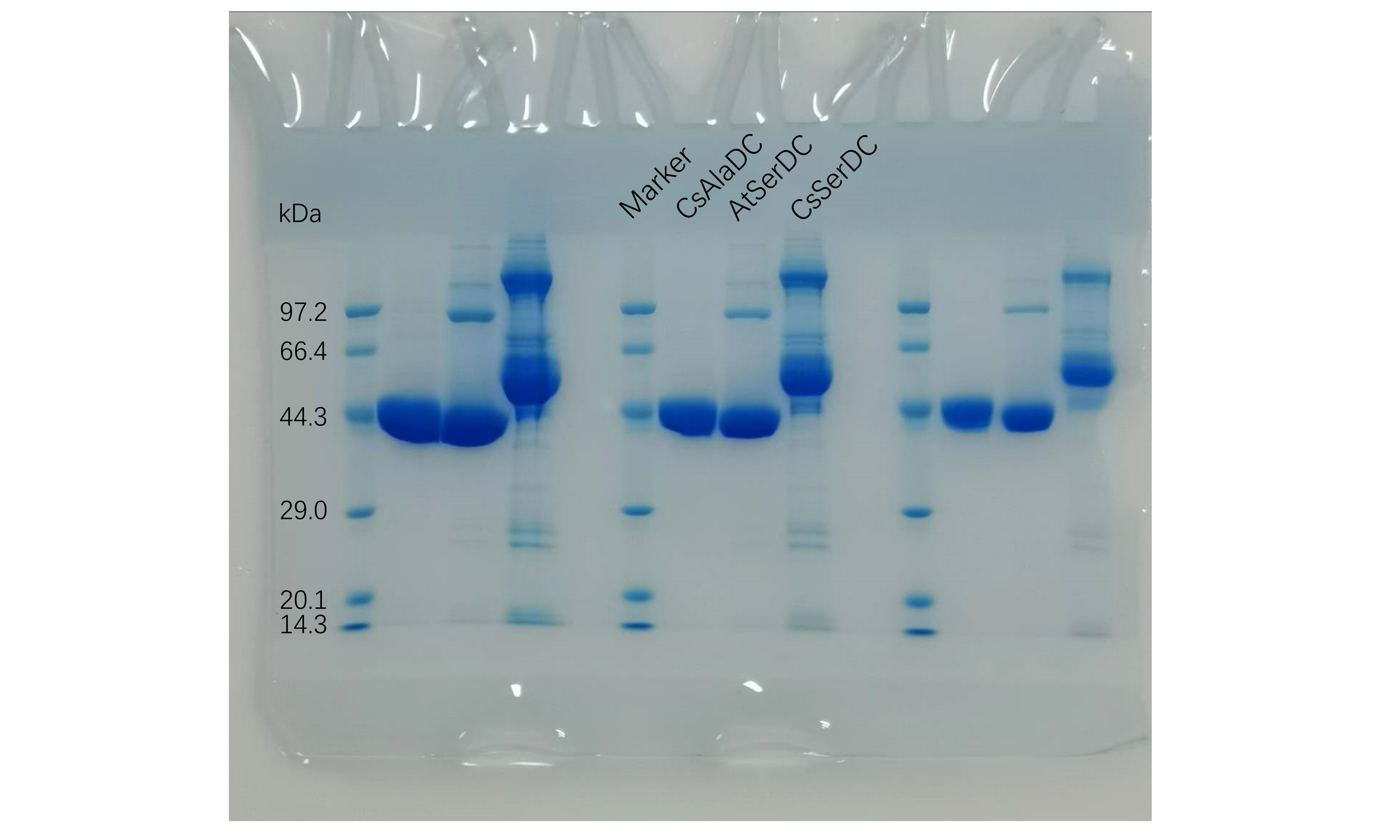

Supplement: Figure 2—source data 1. [file elife-91046-fig2-data1.zip › Figure 2 - Source data 1. Uncropped and labelled gels for Figure 2.png]

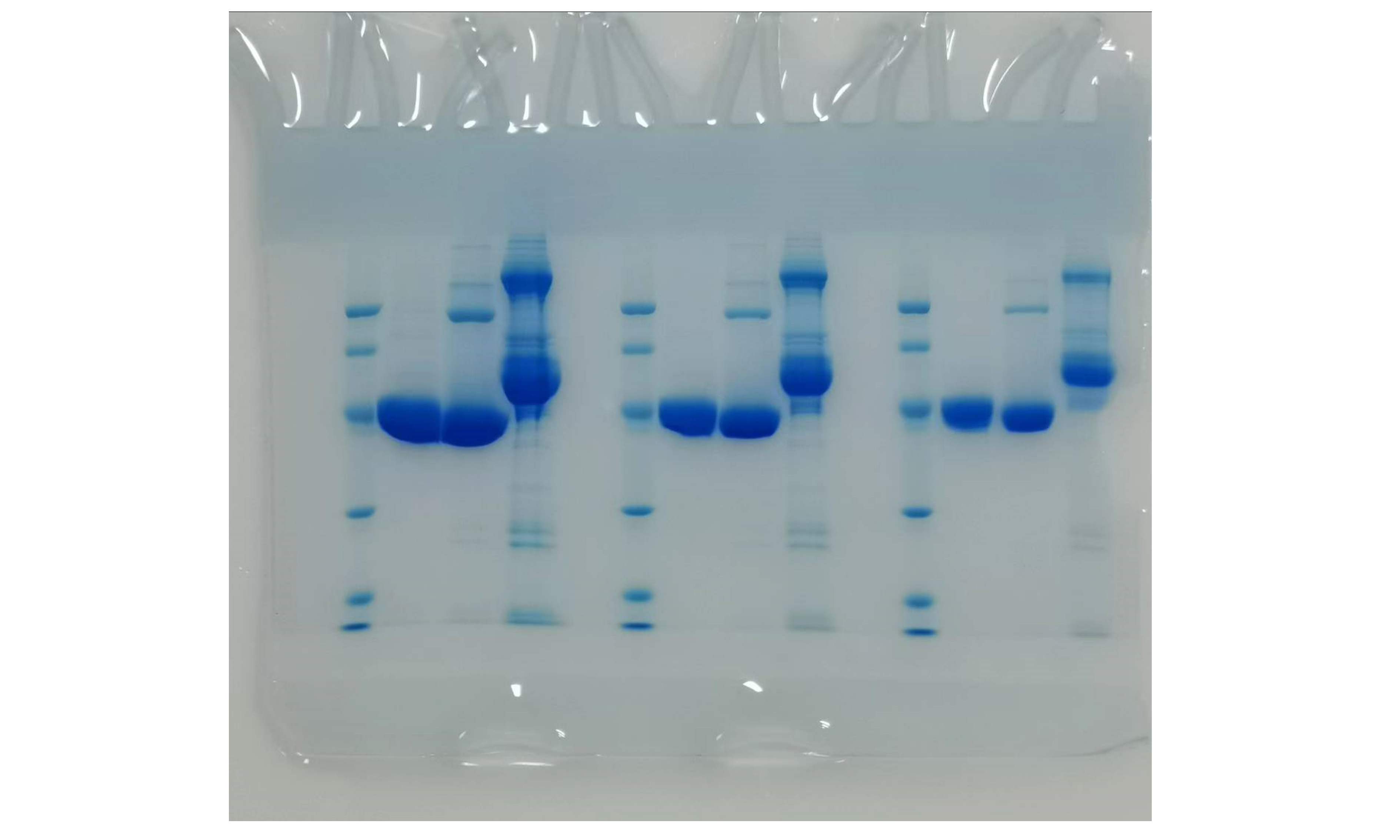

Supplement: Figure 2—source data 2. [file elife-91046-fig2-data2.zip › Figure 2-Source Date 2. Raw unedited gels for Figure 2 .png]

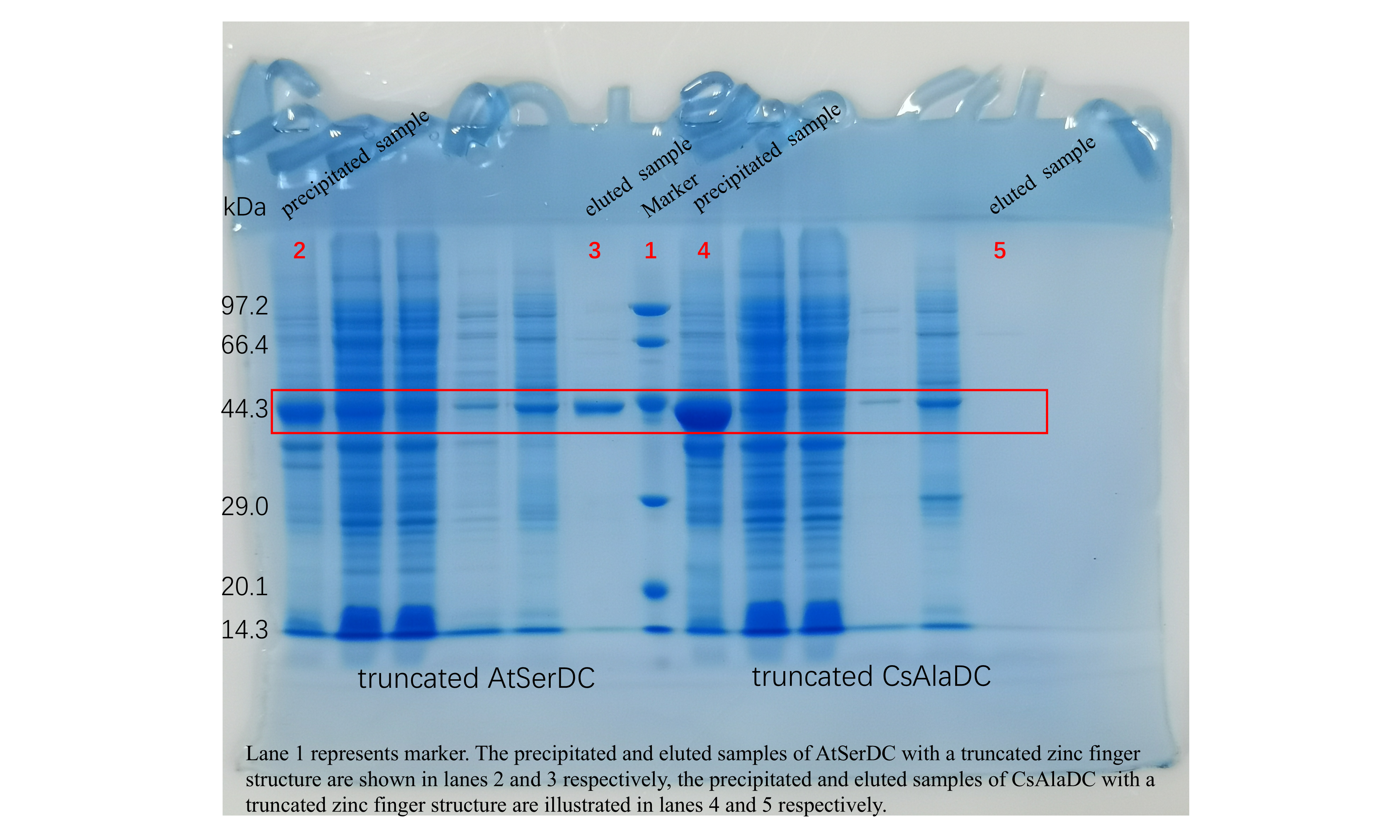

Supplement: Figure 2—figure supplement 2—source data 1. [file elife-91046-fig2-figsupp2-data1.zip › Figure 2-figure supplement 2-Source Date 1. Uncropped and labelled gels for Figure 2-figure supplement 2.png]

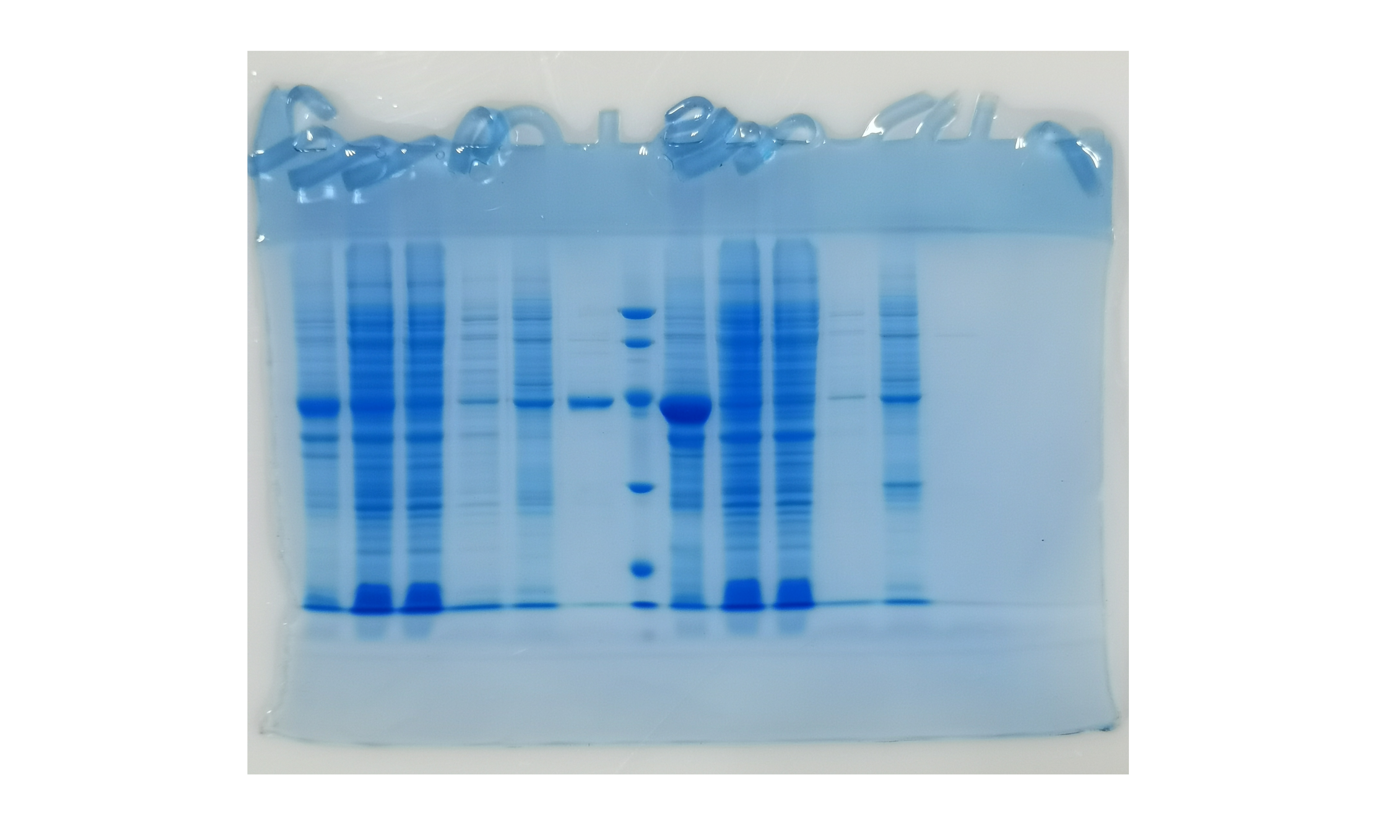

Supplement: Figure 2—figure supplement 2—source data 2. [file elife-91046-fig2-figsupp2-data2.zip › Figure 2-figure supplement 2-Source Date 2. Raw unedited gels for Figure 2-figure supplement 2.png]
