# Supplementary material for: Structure and evolution of alanine/serine decarboxylases and the engineering of theanine production
Source: eLife. 2024 Sep 17;12:RP91046. doi: 10.7554/eLife.91046 (PMC11407765; doi:10.7554/eLife.91046)
Supplement: Supplementary file 1. — (a) Data collection and refinement statistics. (b) Primers used for gene cloning. (c) Primers used for real-time PCR. [file elife-91046-supp1.docx]

**Supplementary** **File** **1a**. Data collection and refinement statistics

|  | CsAlaDC | CsAlaDC-EA complex | AtSerDC |
| --- | --- | --- | --- |
| **Data Collection** |  |  |  |
| Space Group | *P3121* | *P3121* | *C2* |
| Cell Dimensions |  |  |  |
| *a, b, c* (Å) | 163.5,163.5, 50.2 | 164.0,164.0,50.2 | 115.4,149.5,139.1 |
| α, β, γ (°) | α=β=90, γ=120 | α=β=90, γ=120 | α=γ=90, β=109.2 |
| Wavelength (Å) | 0.979 | 0.979 | 0.979 |
| Resolution (Å) | 50-2.50  (2.54-2.50) | 50-2.60  (2.64-2.60) | 50-2.85  (2.90-2.85) |
| Completeness (%) | 99.3 (99.9) | 99.6 (100.0) | 99.6 (98.1) |
| Redundancy | 5.4 (5.1) | 13.0 (12.5) | 5.9 (5.0) |
| *<I>/<σI>* | 9.3 (1.9) | 18.9 (4.6) | 10.6 (2) |
| *R*_merge_ | 0.128 (0.603) | 0.156 (0.562) | 0.173 (0.551) |
| **Refinement** |  |  |  |
| *R*_work_/ *R*_free_ | 0.173/0.222 | 0.172/0.223 | 0.214/0.262 |
| No. reflections | 25175 | 22813 | 49543 |
| B-facator (Å^2^) |  |  |  |
| Protein | 43.3 | 48.3 | 57.6 |
| PLP | 29.5 | 31.5 | 52.8 |
| Zn^2+^ | 58.7 | 43.8 | 71.6 |
| Ca^2+^ | - | 55.5 | - |
| Ethylamine | - | 27.5 | - |
| Glycerol | - | - | 51.7 |
| Water | 38.5 | 40.9 | - |
| R.m.s. deviations |  |  |  |
| Bond lengths (Å) | 0.008 | 0.008 | 0.007 |
| Bond angles (°) | 1.531 | 1.601 | 1.410 |
| No. of atoms |  |  |  |
| Protein | 3292 | 3265 | 3250 |
| PLP | 15 | 15 | 60 |
| Zn^2+^ | 2 | 1 | 4 |
| Ca^2+^ | - | 1 | - |
| Ethylamine | - | 5 | - |
| Glycerol | - | - | 24 |
| Water | 115 | 60 | - |
| Ramachandran statistics (%) |  |  |  |
| Favoured | 94.01 | 93.58 | 91.52 |
| Allowed | 5.74 | 5.68 | 7.61 |
| Outliers | 0.25 | 0.74 | 0.87 |

*R*_merge_=∑hkl∑i|Ii(hkl)-<I(hkl)>|/∑hkl∑i Ii(hkl), where<I(hkl)>is the main value of I(hkl).

*R*_work_=∑||Fobs|-|Fcalc||/∑|Fobs|, where Fobs and Fcalc are observed and calculated structure factors.

The free R factor was calculated using 5% of reflections ommitted from the refinement.

Highest resolution shell is shown in parenthesis

**Supplementary** **File 1b.** Primers used for gene cloning.

| Name | Forward primers (5'-3') | Reverse primers (5'-3') |
| --- | --- | --- |
| *CsAlaDC* | GGAATTCCATATGACAACAAGCCTCACCATCACGG | GATCTCGAGTCATTTATGAAGATCACAATCACAATTCTCACTTC |
| *AtSerDC* | GGAATTCCATATGACCACCAGCCTGGCCG | GATCTCGAGTTATTTATGGGCCGGACAAATGCAATTATTG |
| *CsSerDC* | ATGGTGGGAAGTGTTGGGGT | TCACTTGTGTAGTGCACAAAGACAGT |

**Supplementary** **File 1c.** Primers used for real-time PCR.

| Name | Forward primers (5'-3') | Reverse primers (5'-3') |
| --- | --- | --- |
| *CsAlaDC* | CACTGTGATGGGGCTCTGTT | TGTTATCTGGACACCGCACG |
| *AtSerDC* | TCACTCGCTGTAACGGAACC | AACTGACCAAGCGCACCATA |
| *NbGAPDH* | AGCTCAAGGGAATTCTCGATG | AACCTTAACCATGTCATCTCCC |
